# Humoral and Cellular Immune Responses of Solid Organ Transplant Patients on Belatacept to Three Doses of mRNA-Based Anti-SARS-CoV-2 Vaccine

**DOI:** 10.3390/vaccines10030354

**Published:** 2022-02-24

**Authors:** Florence Abravanel, Olivier Marion, Arnaud Del Bello, Thomas Beunon, Raphaelle Romieu-Mourez, Chloé Couat, Mélanie Pucelle, Laetitia Staes, Joelle Guitard, Laure Esposito, Stanislas Faguer, Nassim Kamar, Jacques Izopet

**Affiliations:** 1 Laboratory of Virology, Toulouse Purpan University Hospital, 31073 Toulouse, France; thomas.beunon@univ-tlse3.fr (T.B.); raphaelle.romieu-mourez@inserm.fr (R.R.-M.); pucelle.m@chu-toulouse.fr (M.P.); staes.l@chu-toulouse.fr (L.S.); izopet.j@chu-toulouse.fr (J.I.); 2 INFINITY—Inserm U1291-CNRS U5051, 31073 Toulouse, France; marion.o@chu-toulouse.fr (O.M.); kamar.n@chu-toulouse.fr (N.K.); 3 School of Medicine Rangueil, Paul Sabatier University, 31062 Toulouse, France; delbello.a@chu-toulouse.fr (A.D.B.); faguer.s@chu-toulouse.fr (S.F.); 4 Department of Nephrology and Organ Transplantation, Toulouse Rangueil University Hospital, 31073 Toulouse, France; couat.c@chu-toulouse.fr (C.C.); guitard.j@chu-toulouse.fr (J.G.); esposito.l@chu-toulouse.fr (L.E.)

**Keywords:** IgG SARS-CoV-2, anti-spike, solid organ transplant recipients

## Abstract

Background: Two doses of anti-SARS-CoV-2 mRNA-based vaccines are poorly immunogenic in solid organ transplant recipients (SOT). Methods: In total, 68 belatacept-treated SOT recipients followed at the Toulouse University Hospital were investigated. They were given three injections of the BNT162b2 mRNA COVID-19 vaccine. Their humoral response was assessed by determining anti-spike antibodies and neutralizing antibodies. The T-cell responses were assessed using an enzyme-linked immunospot assay that measured the interferon-γ produced by specific SARS-CoV-2 T-cells in a subgroup of 17 patients. Results: Only 23.5% of these patients developed a detectable anti-spike response. Moreover, the cellular and the humoral responses were well correlated. Patients with no humoral response were also without a detectable cellular response. Those belatacept-treated patients who developed an Anti-SARS-CoV-2 humoral response were younger, had been transplanted for longer, and had a higher lymphocyte count and a better glomerular filtration rate than those with no response. Finally, patients on tacrolimus plus belatacept produced a lower immune response. Conclusions: Belatacept-treated SOT recipients have a reduced immune response to anti-SARS-CoV-2 mRNA vaccination. The vaccine should be given quite separately from the belatacept infusion to improve immunogenicity. Studies to assess whether switching to another immunosuppressive regimen can improve the post-vaccination immune response would be useful.

## 1. Introduction

Solid organ transplant (SOT) recipients are extremely vulnerable to COVID-19 and their morbidity and mortality rates are higher than those of the general population [1]. As two doses of anti-SARS-CoV-2 mRNA-based vaccines are only poorly immunogenic in these patients, several countries now recommend that they be given a third dose of vaccine [2,3]. Anti-SARS-CoV-2 antibodies were detected in up to 70% of SOT patients given three doses of mRNA-based vaccines [4,5,6]. Their immunosuppressive regimen is the only modifiable factor controlling the humoral response of these patients to anti-SARS-CoV-2 vaccines [6,7].

The CD80/86-CD28 co-stimulation blocker belatacept was approved for use in de novo kidney transplantation in 2011. It has since been shown to be valuable for maintaining immunosuppression [8]. However, the use of belatacept is associated with a poor humoral response to two or three doses of anti-SARS-CoV-2 mRNA vaccines [6,7]. Few data are available on the impact of belatacept on the cellular immune response to anti-SARS-CoV-2 mRNA vaccination. The B-cell and T-cell responses of a cohort of belatacept-treated transplant recipients given three doses of the BNT162b2 mRNA vaccine were therefore examined so as to better characterize the immune response of vaccinated belatacept-treated transplant recipients.

## 2. Materials and Methods

A cohort of 68 belatacept-treated SOT recipients were followed at the Toulouse University Hospital. They were given three intramuscular injections of BNT162b2 mRNA COVID-19 vaccine (Pfizer-BioNTech) (30 µg) between 7 January and 30 August 2021. The first two doses were given one month apart; the third dose was administered 56 ± 11 days after the second dose, as recommended in France [9]. The mRNA vaccine was given between two doses of belatacept, i.e., two weeks after the last belatacept infusion. The controls were 20 healthy volunteers (median age, 55 years) sampled one month after two doses of BNT162b2 vaccine. Ethical approval was obtained from the institutional review board at Toulouse University Hospital (RC31/21/0154).

### 2.1. Serological Assays

Total antibodies to SARS-CoV-2 spike protein were assessed using a serum total SARS-CoV-2 antibody ELISA kit (Beijing Wantai Biological Pharmacy Ent Co., Ltd., Bejing, China) according to the manufacturer’s instructions.

Neutralizing antibody titers were determined using a live virus neutralization assay and a clinical SARS-CoV-2 strain (GISAID: EPI_ISL_804378, GISAID Clade:GH, Pango lineage: B.1.160, Nextclade: 20A.EU2) infecting Vero cells (ATCC, CCL-81^TM^). Briefly, 10^4^ cells were mixed with the virus suspension (100 TCID50) and serial twofold dilutions of the test sera and incubated for 4 days in the wells of 96-well plates. The wells showing a cytopathic effect (CPE) were counted and the titer, the reciprocal of the greatest serum dilution protecting cells, was calculated.

### 2.2. Elispot Assay

T-cell responses were assessed on the day of each injection and 1 month after the third dose using an enzyme-linked immunospot assay (EliSpot) that measured the interferon-γ produced by specific SARS-CoV-2 T-cells. Frozen peripheral blood mononuclear cells were stimulated with individual 15-mers 11-aa overlapping peptide pools derived from a peptide scan through the SARS-CoV-2 spike glycoprotein (two pools representing the spike protein S1 and S2 domains) (JPT-Peptide-Technologies). Results are expressed as spot-forming units (SFU)/10^6^ cells. Negative control wells lacked peptides, and positive control wells included CD3/CD28 and CEF pool stimulation. An IFN gamma response was considered to be positive if the SFU count was >30 SFU/10^6^ cells.

### 2.3. Statistical Analyses

Continuous variables are shown as means (±SEM) or medians (IQR 25–75). Proportions were compared by Fisher’s exact test. Quantitative variables were compared with the Mann–Whitney U-test. A *p*-value of < 0.05 was considered to be statistically significant. Data were analyzed using GraphPad Prism version 9.0.2 (GraphPad Software, San Diego, CA, USA).

## 3. Results

### 3.1. Humoral Response

The 68 belatacept-treated patients (49 men (72%), median age: 61 (52–69) years) included 62 kidney transplant and 6 heart transplant recipients. The median time between transplantation and vaccination was 69 months (interquartile range (IQR) 33–108). Belatacept was given with mycophenolic acid (*n* = 40, 58.8%), mammalian target of rapamycin (mTOR) inhibitors (*n* = 18, 26.5%), tacrolimus (*n* = 18, 26.5%), azathioprine (*n* = 2, 2.9%), and steroids (*n* = 61, 89.7%). No patient had a history of COVID-19.

**Figure 1:**
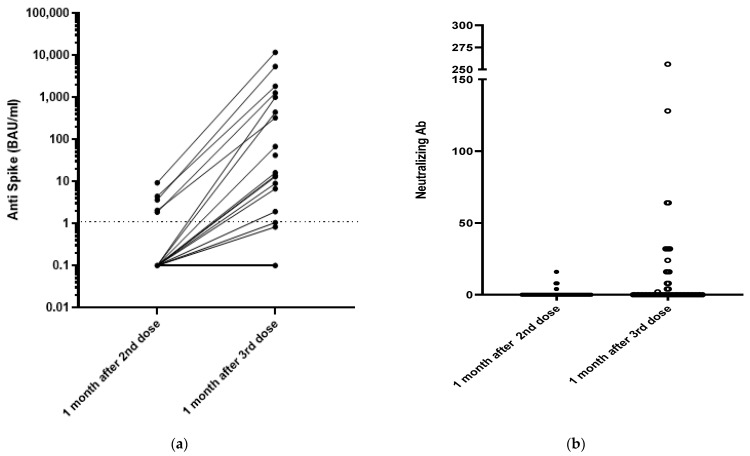
Humoral responses 1 month after the second and the third dose of the mRNA-1273 vaccine. (**a**) Anti-S results using the Wantai assay and (**b**) neutralizing antibodies results.

All the patients were SARS-CoV-2 seronegative at the time of their first vaccination. The Wantai assay indicated that none of the patients developed anti-spike protein antibodies after one dose. Only 5/68 patients (7.4%) developed anti-spike antibodies one month after the second dose. Finally, 16/68 belatacept-treated patients (23.5%) developed anti-spike antibodies 4 weeks after their third dose, with a median antibody titer of 42 (7–1200) BAU/mL and a median neutralizing antibody titer of 24 IU/mL (8–56) (Figure 1). Only seven patients (10%) developed antibody titers greater than 141 BAU/mL, a concentration that provides 89.3% protection in immunocompetent patients [10]. The antibody titers of the five patients who were seropositive after the second dose increased from 2.8 (1.8–9.3) to 402 (87–508) BAU/mL 4 weeks after the third dose (*p* = 0.02). Their neutralizing antibody titers increased from 8 (2–32) to 16 (4–32) (*p* = 0.09).

Only 11 (17.5%) of the 63 patients who were seronegative before their third dose became seropositive (median titer 13 (1.7–658) BAU/mL) 4 weeks after their third dose. Their median neutralizing antibody titer was 16 (2–64).

The antibody responses of the belatacept-treated patients appeared to be lower than those of the 20 healthy controls. The antibody concentrations of the controls 1 month after two doses of the BNT162b2 vaccine were all >141 BAU/mL (median 1309 (457–7605) BAU/mL); their neutralizing antibody titers were all >64 IU/mL (range: 64 to 512 IU/mL).

### 3.2. T-Cell Response

**Figure 2:**
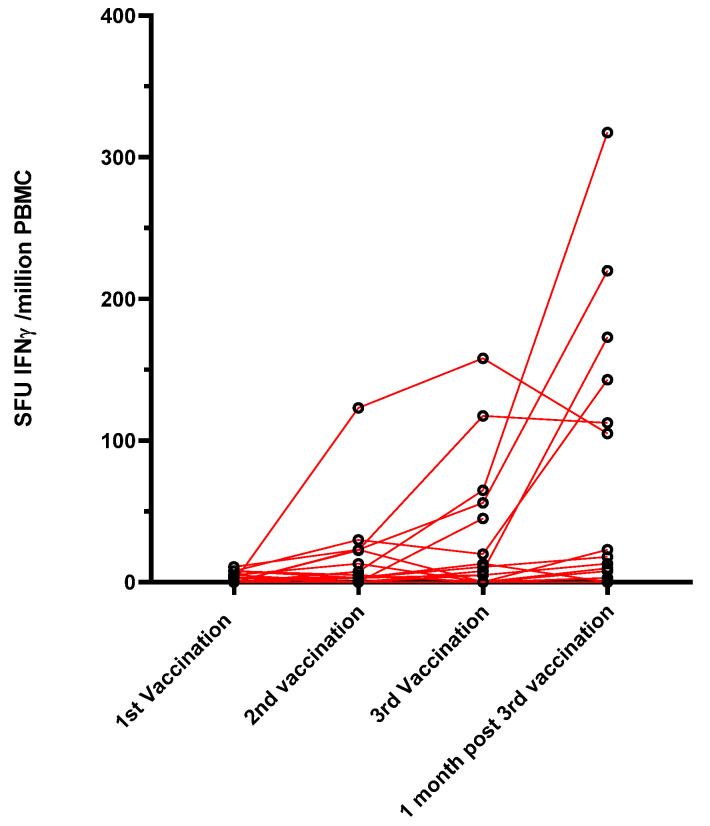
Evolution of the cellular response using an ELISpot assay against S peptides (SFU: spot-forming unit).

The specific T-cell response to the SARS-CoV-2 spike protein was investigated in a subgroup of 17 belatacept-treated patients, for whom enough PBMC were collected, using an Elispot assay that detects the interferon gamma response against pools of S peptides. None of the patients had a specific T-cell response before vaccination. Only 2/17 (12%) patients produced a low specific T-cell response one month after the first dose, 5/17 patients (29.4%) had a T-cell response one month after the second dose, and 6/15 (40%) had a T-cell response one month after the third dose (Figure 2).

**Figure 3:**
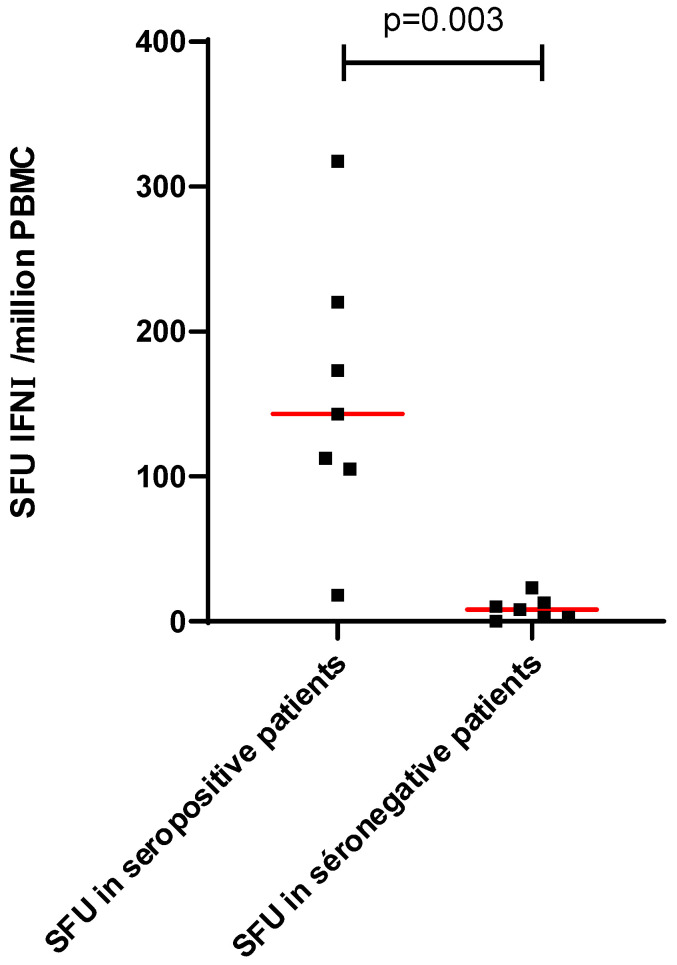
Cellular response according to the seroconversion 1 month after the third dose of the mRNA-BNT162b2 vaccine. Red line represents the median.

Patients with no seroconversion 4 weeks after the third dose produced no detectable interferon gamma response to the S1 and S2 peptide pools (Figure 3).

**Figure 4:**
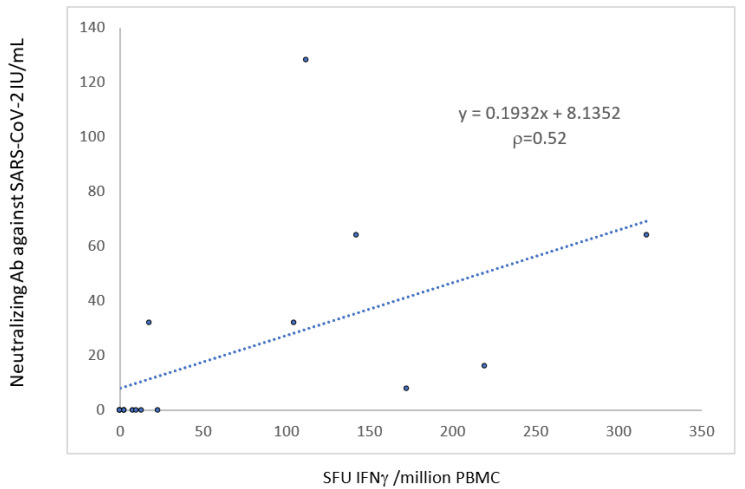
Correlation between the humoral and the cellular anti-S response 1 month after the third dose of the mRNA-BNT162b2 vaccine.

The T-cell response was correlated with the neutralizing antibody response (r = 0.52, *p* < 0.05) (Figure 4). 

### 3.3. Factors Associated with an Immune Response

The SOT recipients who developed anti-SARS-CoV-2 antibodies after vaccination (i.e, responders) were younger and had been transplanted for longer than non-responders (Table 1). No belatacept-treated heart transplant recipient developed post-vaccination antibodies.

**Figure 5:**
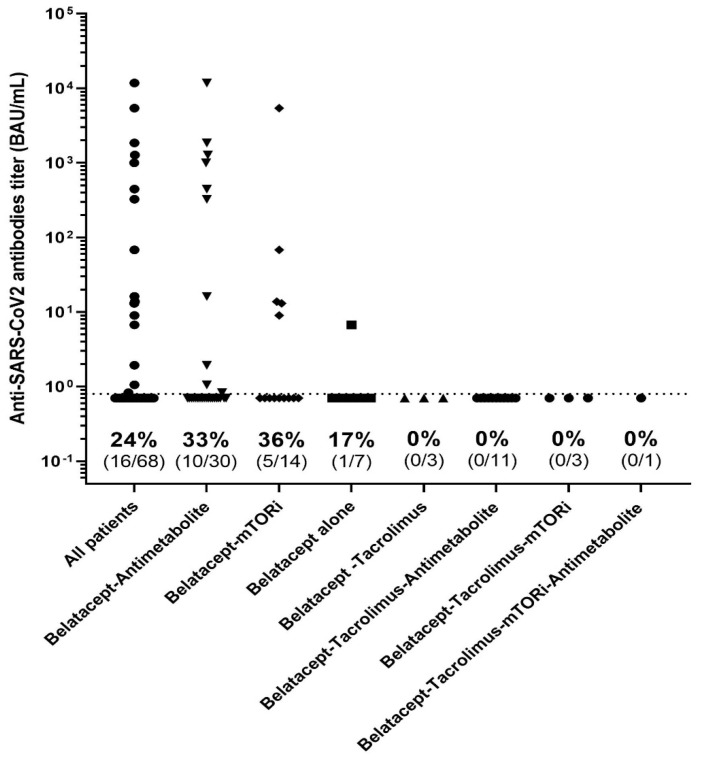
Anti-S results, using the Wantai assay, according to the immunosuppressive regimen.

Those patients given tacrolimus in addition to belatacept had significantly poorer antibody responses to three doses of the vaccine (0% (*n* = 0/18)) than those not on tacrolimus (37.5%, *n* = 16/50, *p* = 0.018). Other combinations of immunosuppressant—belatacept with mTOR inhibitors or mycophenolate acid—had no impact on the humoral immune response after vaccination (Figure 5). Steroid use in responders and non-responders was similar.

SOT recipients treated with belatacept who developed an anti-SARS-CoV-2 immune response had higher lymphocyte counts at baseline (1850 ± 215/mm^3^) than patients with no immune response (1201 ± 90/mm^3^, *p* < 0.01). They had higher CD4^+^ T-cell and CD19^+^ B lymphocyte counts (Table 1). The CD8^+^ T-cell count tended to be higher in responders, but not significantly. Conversely, the NK cell counts in the two groups were similar.

Finally, patients with a humoral response after vaccination had higher estimated glomerular filtration rates (63 ± 7 mL/min/1.73 m^2^) than non-responders (42 ± 3 mL/min/1.73 m^2^, *p* = 0.019).

## 4. Discussion

Belatacept-treated transplant recipients appear to produce low humoral and cellular responses to three doses of the mRNA-BNT162b2 vaccine. Only 23.5% of these patients developed a detectable anti-spike response. Moreover, the cellular and the humoral responses were well correlated. Patients with no humoral response were also without a detectable cellular response. Those belatacept-treated patients who developed an Anti-SARS-CoV-2 humoral response were younger, had been transplanted for longer, and had a higher lymphocyte count and a better glomerular filtration rate than those with no response. Finally, patients on tacrolimus plus belatacept produced a lower immune response.

Overall, 23% (16/68) of the patients had detectable antibodies by using an ELISA assay or neutralizing antibodies. In SOT recipients not treated with belatacept, the seroconversion rate was evaluated between 49% and 70% after three doses of the mRNA vaccine [4,5,6]. In a previously study in SOT recipients followed in our center, the seroconversion rate of the SOT recipients not treated with belatacept was 66.4% after three doses of the mRNA vaccine [6]. In addition, in healthy controls, antibody concentrations at 1 month after two doses of the BNT162b2 vaccine were greater than 140 BAU/mL (median, 1309 BAU/mL (range, 457–7605 BAU/mL)) and NAb titers were greater than 64 IU/mL (median 128 (range, 64–512 IU/mL)) [11]. The cellular responses of the belatacept-treated patients appeared to be lower than those of the 20 healthy controls. The specific T-cell response of the controls 1 month after two doses of the BNT162b2 vaccine was 542 SFUs IFN gamma per 10^6^ PBMCs (range, 0–1669 SFUs per 10^6^ PBMCs) [11].

Ou et al. reported recently that kidney transplant recipients on belatacept were 16.7 times less likely to have a positive antibody response to two doses of mRNA vaccines [12]. Several studies have used an ELISA assay to assess the antibody response of belatacept-treated kidney-transplant recipients given three doses of the mRNA anti-SARS-CoV-2 vaccine [13,14]. Chavarot et al. reported a seroconversion rate of only 6.4% (4/62) [13], while Noble et al. found a seroconversion rate of 20% (4/20) [14], similar to the present results. These differing humoral responses could be due to the timing of the anti-SARS-CoV-2 mRNA vaccinations and the belatacept infusion. We, like Noble et al., vaccinated the patients between two belatacept infusions: on day 21 post-belatacept for Noble et al. and day 14 in this study. Chavarot et al., in contrast, vaccinated their patients on the same day as the belatacept infusion. Nevertheless, the antibody response, expressed in BAU/mL or neutralizing antibodies, was much lower than in immunocompetent patients. Only 10% of belatacept-treated patients developed an antibody titer greater than 141 BAU/mL, a protective threshold [10].

Those of the patients who showed no seroconversion produced no detectable T-cell response, unlike the findings of previous studies on SOT recipients. Several reported positive anti-spike T-cell responses in patients who did not develop detectable anti-spike antibodies [15,16,17]. Cucchiari et al. found that half their seronegative kidney transplant patients had detectable anti-S protein T-cell responses after two doses of the mRNA-1273 vaccine [15]. Herrera et al. obtained similar results in heart and liver transplant recipients; approximately 70% of their seronegative patients developed a T-cell response after two doses of the mRNA-1273 vaccine [16]. Similarly, the belatacept-treated patients examined by Bertrand et al. had no antibody response, but 40% (4/10) of them developed a low T-cell response [18]. The difference in the B- and T-cell responses of belatacept-treated patients to anti-SARS-CoV-2 vaccination could be because the cross-talk between B and Tfh cells is inhibited by the CTLA4, leading to impaired germinal center formation and an improper antibody response [19]. We found no such discrepancy between the T- and B-cell responses to the mRNA-based vaccination in the present larger cohort, perhaps because other immunosuppressants—like mycophenolic acid, mTOR, and calcineurin inhibitors—targeting the T-cell response were frequently used in the present cohort.

The factors associated with a lack of response to three doses of the anti-SARS-CoV-2 vaccine were patient age, recent transplantation, tacrolimus use, an altered eGFR, and a low lymphocyte count. Advanced age has been found to be associated with a poor response to vaccine in both immunocompetent and immunocompromised patients [6,7,20]. SOT recipients are placed on an immunosuppressive regimen to prevent allogenic T-cell activation and the proliferation of activated B and T lymphocytes. Antimetabolites have been associated with a poor humoral response to mRNA vaccination [3,6,7]. The combination of tacrolimus and belatacept had a negative impact on the humoral response of the SOT patients included in this study. This combination appears to be deeply immunosuppressive, and its use should be limited to rescuing patients with a history of rejection and altered graft function [21].

Studies to assess whether switching to another immunosuppressive regimen can improve the post-vaccination immune response would be useful. Other vaccine strategies should also be investigated in this population. Bruminhent et al. have assessed the immune response to an inactivated vaccine [22]. They found a weak humoral response but comparable cellular responses in fully vaccinated kidney transplant recipients receiving the inactivated SARS-CoV-2 vaccination compared to immunocompetent individuals. Additionally, a third vaccine with a higher dose of mRNA and a longer interval between doses of the primary vaccination, such as mRNA-1273, can be an effective alternative [23].

As it was imperative to protect the patients against SARS-CoV-2 infection, 51/68 patients received monoclonal neutralizing antibodies casirivimab-imdevimab as pre-exposure prophylaxis. In total, 1 patient without seroconversion out of the 17 patients that did not receive the prophylaxis developed a severe fatal infection with delta SARS-CoV-2. Additionally, 5 patients out of 51 receiving casirivimab-imdevimab as pre-exposure prophylaxis developed a moderate infection with omicron SARS-CoV-2 (data not shown).

## 5. Conclusions

In conclusion, belatacept-treated SOT recipients have a reduced immune response to anti-SARS-CoV-2 mRNA vaccination. The vaccine should be given quite separately from the belatacept infusion to improve immunogenicity. Other strategies, such as pre- and post-exposure prophylaxis with neutralizing anti-SARS-CoV-2 monoclonal antibodies active on the circulating SARS-C0V-2 variant, have to be considered for poor- or non-responding SOT patients. This strategy was approved by the French Authorities [24].

## Figures and Tables

**Table 1:** Clinical and biological characteristics of solid organ transplant recipients treated with belatacept.

	Responders (*N* = 16)	Non-Responders (*N* = 52)	*p*-Value
Sex ratio (M/F)	4.3 (13/3)	2.3 (36/16)	0.53
Age (years, mean ± SEM)	55 ± 3	63 ± 2	0.02
Type of organ transplant, *n* (%) Kidney Heart			
	16 (100)	46 (88)	0.32
	-	6 (12)	
History of rejection in the year preceding vaccination, *n* (%)	0 (0)	5 (10)	0.33
Time between vaccine and transplantation (months, mean ± SEM)	132 ± 24	80 ± 12	0.01
No induction therapy, *n* (%) Induction therapy, *n* (%) Anti-IL2 receptor Thymoglobulin	5 (31)	22 (42)	0.56
	11 (69)	30 (58)	
	8 (73)	17 (57)	
	3 (27)	13 (43)	
Type of immunosuppressive regimen, *n* (%) Tacrolimus Antimetabolite Mycophenolic acid Azathioprine mTOR inhibitors Steroids			
	0 (0)	18 (35)	<0.01
	10 (63)	32 (61)	1
	9	31	
	1	1	
	5 (31)	13 (25)	0.75
	13 (81)	48 (92)	0.34
Neutrophil count (/mm^3^, mean ± SEM)	5250 ± 509	6009 ± 474	0.55
Lymphocyte count (/mm^3^, mean ± SEM)	1850 ± 215	1201 ± 90	<0.01
CD4+ T-cell count (/mm^3^, mean ± SEM)	*n* = 13	*n* = 32	<0.001
	671 ± 61	325 ± 33	
CD8+ T-cell count (/mm^3^, mean ± SEM)	*n* = 13	*n* = 32	0.10
	634 ± 135	351 ± 37	
CD19+ T-cell count (/mm^3^, mean ± SEM)	*n* = 13	*n* = 33	0.03
	120 ± 27	93 ± 30	
NK cell count (/mm^3^, mean ± SEM)	*n* = 13	*n* = 33	0.53
	284 ± 52	233 ± 29	
eGFR (mL/min/1.73 m^2^) Kidney transplant eGFR Non-kidney transplant eGFR	63 ± 7	42 ± 3	0.02
	63 ± 7	41 ± 3	0.01
	-	53 ± 12	-
Positive anti-SARSCoV2 antibodies before vaccination	0	0	1

Abbreviations: IL2, interleukin 2; mTOR, mammalian target of rapamycin; NK, natural killer; eGFR, estimated glomerular filtration rate (Chronic Kidney Disease Epidemiology Collaboration (CKD-EPI) equation).

## Data Availability

The datasets generated and analyzed during the current study are available from the corresponding author on reasonable request.
